# Production and Comprehension of Prosodic Markers in Sign Language Imperatives

**DOI:** 10.3389/fpsyg.2018.00770

**Published:** 2018-05-23

**Authors:** Diane Brentari, Joshua Falk, Anastasia Giannakidou, Annika Herrmann, Elisabeth Volk, Markus Steinbach

**Affiliations:** ^1^Department of Linguistics, Center for the Study of Gesture, Sign and Language, University of Chicago, Chicago, IL, United States; ^2^Department Language, Literature, Media I, Institute for German Sign Language, University of Hamburg, Hamburg, Germany; ^3^Sign Language Lab and RTG 2070 “Understanding Social Relationships”, Department of German Philology, University of Göttingen, Göttingen, Germany

**Keywords:** imperatives, speech acts, sign languages, gesture, prosody, semantics, non-manual markers

## Abstract

In signed and spoken language sentences, imperative mood and the corresponding speech acts such as for instance, command, permission or advice, can be distinguished by morphosyntactic structures, but also solely by prosodic cues, which are the focus of this paper. These cues can express paralinguistic mental states or grammatical meaning, and we show that in American Sign Language (ASL), they also exhibit the function, scope, and alignment of prosodic, linguistic elements of sign languages. The production and comprehension of prosodic facial expressions and temporal patterns therefore can shed light on how cues are grammaticalized in sign languages. They can also be informative about the formal semantic and pragmatic properties of imperative types not only in ASL, but also more broadly. This paper includes three studies: one of production (Study 1) and two of comprehension (Studies 2 and 3). In Study 1, six prosodic cues are analyzed in production: temporal cues of sign and hold duration, and non-manual cues including tilts of the head, head nods, widening of the eyes, and presence of mouthings. Results of Study 1 show that neutral sentences and commands are well distinguished from each other and from other imperative speech acts via these prosodic cues alone; there is more limited differentiation among explanation, permission, and advice. The comprehension of these five speech acts is investigated in Deaf ASL signers in Study 2, and in three additional groups in Study 3: Deaf signers of German Sign Language (DGS), hearing non-signers from the United States, and hearing non-signers from Germany. Results of Studies 2 and 3 show that the ASL group performs significantly better than the other 3 groups and that all groups perform above chance for all meaning types in comprehension. Language-specific knowledge, therefore, has a significant effect on identifying imperatives based on targeted cues. Command has the most cues associated with it and is the most accurately identified imperative type across groups—indicating, we suggest, its special status as the strongest imperative in terms of addressing the speaker's goals. Our findings support the view that the cues are accessible in their content across groups, but that their language-particular combinatorial possibilities and distribution within sentences provide an advantage to ASL signers in comprehension and attest to their prosodic status.

## Introduction

It is well known that signers use their hands, body, head, and face for both grammatical and gestural purposes (see Goldin-Meadow and Brentari, 2017 for a review), and non-manual markers have been identified in signed and spoken languages to express sentence meaning, as well as emotion, intention, and the mental states of signers and speakers (for sign languages see Baker-Shenk, 1983; Poizner et al., 1987; Emmorey, 1999; Wilbur, 2003; Dachkovsky and Sandler, 2009; Dachkovsky et al., 2013; for good summaries see Pfau and Quer, 2010; Sandler, 2012; for spoken languages see Bolinger, 1983; Borràs-Comes and Prieto, 2011; Borràs-Comes et al., 2014; Domaneschi et al., 2017). In this paper, presenting three studies, we analyze the temporal and non-manual prosodic cues associated with imperative constructions as expressed in American Sign Language (ASL). We argue that, while the non-manuals may be comprehensible to non-signers to a large extent, in a sign language they take on specific distributions as part of sign language prosody and achieve grammatical status (cf. Nespor and Sandler, 1999; Wilbur, 1999, 2009, 2011, 2018; Sandler and Lillo-Martin, 2006; Brentari et al., 2011, 2015; Sandler, 2012).

Our motivation for this group of studies is 2-fold. First, we want to better interpret how signers and non-signers understand the prosodic cues of a sign language in the expression of speech acts, especially non-manuals that may also be comprehensible by non-signers to some extent (for prosodic cues of speech acts in spoken languages see Hellbernd and Sammler, 2016). Because of their pragmatic status as directive speech acts, imperatives engage a number of expressive facial expressions that have this intermediate status. To address this question we carefully annotate and analyze the cues produced in ASL imperatives in a production study (Study 1), and we then use those productions as stimuli for two comprehension studies (Studies 2 and 3), which include two groups of Deaf signers—ASL and German Sign Language (DGS)—as well as two hearing groups of non-signers from the United States and Germany. If all groups are able to perform equally well on a task of imperative comprehension, this would lead us to conclude that the prosodic cues and their patterns in ASL are equally accessible to signers and non-signers, while any differences among the groups would allow us to infer that modality- and language-specific experience, or effects of a specific gestural competence within a community, can affect accessibility. A second motivation for these studies is to shed light on the semantics that underlie imperative types (i.e., the imperative sentence mood). We investigate which imperative speech acts are most clearly distinguished in production, and which are most easily comprehensible across groups.

### Sign language prosody

Prosody in sign languages takes the form of temporal properties of a word or phrase and accompanying non-manual cues. As in spoken languages, sign language prosody is relevant at several grammatical levels. At the lexical level, Wilbur (1999) has argued that the telicity of verbs and phrasal prominence are expressed by the prosodic properties of acceleration and deceleration, and this type of prominence can be paired with a number of different sign types. The temporal markers of sign language prosody, such as lengthening a sign's duration or final hold together with non-manual edge markers such as head nods and eye blinks have been argued to mark constituent boundaries (cf. Nespor and Sandler, 1999; Sandler, 1999; Brentari et al., 2011, 2015).

Linguistic and gestural descriptions of non-manual markers refer to aspects such as position, movement, tension, aperture, and duration of musculature of the face, the head, and the body. Grammatical non-manual markers combine simultaneously with manual components as well as with other non-manual markers; that is, the grammatical information marked by non-manuals can be layered in a complex fashion (cf. Wilbur, 2000; Dachkovsky and Sandler, 2009). Non-manuals are also capable of deriving compositional meaning (cf. Nespor and Sandler, 1999; Sandler and Lillo-Martin, 2006; Herrmann, 2015). Sandler and Lillo-Martin (2006) and Dachkovsky and Sandler (2009) demonstrated that factual conditional sentences in Israeli Sign Language (ISL) are marked by brow raise, whereas counterfactual conditionals require an additional squint. They argue that each non-manual marker has inherent semantic properties, which are compositionally combined to derive the more complex counterfactual meaning. In other cases, the layering of non-manual markers is ascribed to the strong physical relation of the specific components, which jointly fulfill the same grammatical function. This applies, for example, to the forward head tilt in polar questions of DGS, which is regularly accompanied by a forward body lean (cf. Herrmann and Pendzich, 2014). A further distinction is commonly drawn between the upper face and the lower face among grammatical non-manuals (cf. Liddell, 1980; Coerts, 1992; Wilbur, 2000) whereby the upper face includes movements of the eyes and brows, and has been grammatically associated with larger units of prosody (phrases, clauses, utterances) whereas movements and positions of the lower face and mouth have been associated with smaller prosodic units, such as the syllable and prosodic word (Wilbur, 2000; Brentari and Crossley, 2002). In the studies we present here, the focus is on the eyes, head, and the presence or absence of “mouthings,” which are the partial, silent articulations of an English word.

With regard to the grammatical structure of sign languages, non-manual markers play an essential role at all levels of grammar. Starting with phonology, non-manual markers can be lexically specified representing an inherent phonological feature of an individual sign (cf. Coerts, 1992; Brentari, 1998; Woll, 2001; Liddell, 2003; Pfau and Quer, 2010; Pendzich, 2016). The sign recently in DGS is, for instance, produced with a slightly protruded tip of the tongue (cf. Herrmann and Pendzich, 2014). The sign recently in ASL requires a subtle sideward head turn and tensed cheek muscles (cf. Liddell, 1980). Moreover, non-manual markers operate on the level of morphology expressing adverbial and adjectival meanings (cf. Liddell, 1980; Vogt-Svendsen, 2001; Pfau and Quer, 2010). A specific non-manual configuration in ASL using a tongue-thrust functions as an adverbial modifier meaning *carelessly* (cf. Liddell, 1980, p. 50).

Non-manual markers can also affect phrasal and sentence meaning and spread over larger prosodic domains such as phonological and intonational phrases (cf. Sandler, 2010; Crasborn and van der Kooij, 2013; Herrmann, 2015; among others).The prosodic component is autonomous in the grammar and has been shown to interface with the semantics and pragmatics of sign languages (Sandler and Lillo-Martin, 2006; Sandler, 2010). While non-manuals can indicate syntactic constituency when combined with certain types of signs, as in the case of relative clauses in ASL (Liddell, 1980), the timing and spreading behavior of non-manual markers is associated with prosodic constituency, in particular with the intonational phrase (cf. Nespor and Sandler, 1999; Sandler, 2010). As demonstrated in (1) from ISL (Meir and Sandler, 2008, p. 153), the intonational phrase is not necessarily isomorphic with the syntactic domain. The whole sentence represents a polar question syntactically, but the non-manual marker *brow raise* (br) is argued to correspond to a rising question intonation, which only scopes over the first conjunct (cf. Sandler, 2010).

1. The prosodic, rather than syntactic domains of prosodic cues in ISL (Meir and Sandler, 2008)
  __________________________________br
  you want ice cream white ix-a or chocolate ix-b [ISL]
  ‘Do you want vanilla ice cream or chocolate?'

In addition to grammatical non-manuals, such as those described above, both spoken and signed communication also involve facial expressions to express affective meanings and mental states (cf. Campbell et al., 1999; Keltner et al., 2003; McCullough et al., 2005), which we will refer to as “expressives.” Several affective facial expressions associated with a set of basic emotional states such as anger, sadness, or joy are claimed to be universal and therefore cross-culturally conveyed in a similar way (cf. Ekman and Friesen, 1971; Izard, 1994; Benitez-Quiroz et al., 2016). Affective facial expressions include several types, however. One type involves evaluative meaning as the expression of mental states, such as “surprise” or “puzzlement” (cf. Campbell, 1997; Emmorey, 1999).

Another important group of gestural non-manual markers are “iconic” and mimetic mouth gestures (cf. Sandler, 2009). Some iconic mouth gestures are comparable to manual iconic co-speech gestures (cf. McNeill, 1992), since they are produced simultaneously with signs and convey properties of objects or events. Accordingly, Sandler (2009) demonstrates that iconic mouth gestures can be used in narration settings to embellish or complement the linguistic descriptions produced by the hands. In her study of ISL, signers used a manual classifier construction to depict the journey of a cat up a drainpipe, while one of several ways to indicate the narrowness of the pipe was a tightened mouth movement, and one of several ways to indicate a bend in the drainpipe was a zigzag mouth movement, which aligned with the manual linguistic descriptions. These iconic forms were variable across signers and this was one of the reasons for considering them gestural. We consider expressive and iconic forms to be different types of non-manuals. Both may be accessible to non-signers to some degree, but the former refers to the speaker's or signer's affect (or quoted speaker's or signer's affect). Iconic forms refer to the properties of an entity (e.g., size, shape) or an event (manner).

Even though grammatical and affective non-manuals share the same articulatory bases, they are argued to differ in a number of important ways. Experimental evidence has been helpful in distinguishing grammatical and affective non-manual markers. For instance, in grammaticality judgment tasks, signers have clear intuitions about grammatical non-manuals. By contrast, affective non-manuals result in greater within-individual variability (cf. Baker-Shenk, 1983; Poizner et al., 1987; Emmorey, 1999; Wilbur, 2003). In addition, McCullough and Emmorey (2009) investigated whether stimuli of continuously varying facial expressions are perceived categorically, i.e., whether they result in categorical perception (CP) effects. They found that sign language experience influences CP effects for grammatical, but not affective, non-manuals. Further evidence for distinctive representations of grammatical and affective non-manuals is based on neuropsychological studies, which demonstrate that grammatical facial expressions are processed in the left hemisphere, whereas affective facial expressions activate areas in the right hemisphere of the brain (cf. Poizner et al., 1987; Corina et al., 1999; McCullough et al., 2005; Corina and Spotswood, 2012). Finally, research on sign language acquisition reveals that Deaf infants are competent in using a set of affective non-manual markers such as the side-to-side headshake or brow furrow in both production and perception at an early age, but their grammatical use appears later during acquisition. Moreover, when both a manual and non-manual marker have the same grammatical function, such as in conditionals in ASL, the manual marker is acquired first (cf. Baker-Shenk, 1983; Reilly et al., 1990; Emmorey et al., 1995; Morgan and Woll, 2002; Reilly and Anderson, 2002; Brentari et al., 2015).

Grammatical and affective non-manuals differ in their distribution in terms of on- and offset, scope, and apex (cf. Liddell, 1980; Corina et al., 1999; Wilbur, 2003; Dachkovsky, 2007). Accordingly, the on- and off-set of grammatical non-manual markers align with consistent phrasal boundaries, and grammatical non-manuals display a sudden increase of intensity and have an abrupt onset and offset. In other words, the scope and source of grammatical non-manuals are linguistically defined. In contrast, non-manuals expressing emotional and evaluative states that do not contribute to the linguistic meaning display a gradual on- and offset as well as more variable spreading behavior (cf. de Vos et al., 2009), and the apex of intensity also allows for more variability (cf. Liddell, 1980).

One widely held view about non-manual marking is that emotional and mental states, iconic depictions, and discursive functions may be more accessible to non-signers, while grammatical markers may be more arbitrary and inaccessible (cf. Herrmann and Pendzich, 2014). However, even grammatical facial expressions in sign languages have varying degrees of accessibility, as seen in examples (2) and (3)—ranging from those largely accessible to non-signers [e.g., head nod to mean positive assertion (2a) vs. headshake to mean negation (2b)] to those that are relatively inaccessible (e.g., conditionals; see also Malaia and Wilbur, 2012; Malaia et al., 2013; Strickland et al., 2015). Even if negative headshake has language-particular distributional properties that are relevant for the syntactic, typological groups of sign languages (cf. Quer, 2012), both head nods for assertion and headshake for negation are also accessible to non-signers. In contrast, the difference between two simple conjoined clauses in (3a) and a complex conditional construction in (3b) is thought to be less accessible to non-signers. Both clauses have neutral expressions, but in (3a), the neutral expression is extended over both clauses, while in (3b), the first clause has a brow raise used for conditionals. These four sentences are included in the Supplementary Materials.

(2) Sentence meanings that are relatively accessible to non-signers
  a. Assertion (Video 1, Supplementary Materials)
    head nod
    i    go
    “I'm going.”
  b. Negation (Video 2, Supplementary Materials)
    headshake
    i    go
    “I'm not going.”

(3) Sentence meanings that are relatively inaccessible to non-signers
  a. Coordinate Structure (Video 3, Supplementary Materials)
    _________________________neutral
    you come    i leave
    “You come and I'll leave.”
  b. Conditional Structure (Video 4, Supplementary Materials)
    __________*brow raise*__________*neutral*
    you come    i leave
    “If you come, I'll leave.”

This investigation targets the moment at which affective/expressive forms take on systematic linguistic distributions. As expressives, they may only be *paralinguistic* (Bolinger, 1983), and if that is the case there should be no advantage for knowing the grammar of ASL; however, if they have a systematic function, scope, and alignment in production and are used to the advantage of the ASL signers, we have evidence for their systematically linguistic status as part of the prosodic system. We will argue that the temporal and non-manual properties of the expressives associated with imperatives that we investigate in this paper are grammatical and prosodic, even though they may be accessible to non-signers, since they scope over and align with specific phrases, add prominence and also suprasegmental meaning, and have a semantic and syntactic role as well.

### Imperatives

We now turn to the semantic and pragmatic properties of imperatives. Following recent analyses of imperatives (Portner, 2007; Condoravdi and Lauer, 2012; Kaufmann, 2012; von Fintel and Iatridou, 2017), we assume that imperative sentence types are associated with a conventionalized meaning, the “imperative mood.” The imperative meaning appears to be flexible, and is compatible with a range of speech acts such as for example, command, warning, and permission. Across languages, both spoken and signed, imperatives employ prosodic cues along with lexical and morphological markers, such as particles, word order or verbal inflection, and imperatives can also be expressed by prosodic cues alone (see Iatridou, 2008; Hellbernd and Sammler, 2016; von Fintel and Iatridou, 2017 for spoken languages; Donati et al., 2017 for sign languages). Donati et al. (2017) present an in-depth study of imperatives in three sign languages—Italian Sign Language (LIS), French Sign Language (LSF), and Catalan Sign Language (LSC). They found that a number of manual signs, as well as temporal and non-manual markers were used to express different types of imperatives cross-linguistically. While there are more than four pragmatic types of imperatives studied in Donati et al. (2017), in the current studies we focus on the four speech acts expressed by the imperatives described in (4). As we discuss below, these four speech acts belong to the group of illocutionary forces typically realized with imperatives. At the same time, the contextual conditions on these four speech acts are different enough to clearly distinguish these speech acts from each other^1^.

(4) Imperatives and example contexts
  a. Commands: You must do ‘x’.
        Possible context: You and a friend are in a library and you are trying to hurry your friend along. You say, “*Find a book*, and let’s go.”
  b. Explanation: You must do ‘x’ in order to achieve some goal.
        Possible context: You and a friend are in a library and you are explaining how to borrow a book. You say, “*Find a book*, take it to the desk, show your card, and allow them to stamp the book with the due date.”
  c. Permission: You may do ‘x’.
        Possible context: You and a friend are in a library and you agree to allow her to borrow a book with your library card, since she does not have one. You say, “*Find a book* and use my card.”
  d. Advice: You ought to or may do ‘x’ if you want to achieve some goal.
        Possible context: You and a friend are in a library and your friend asks for advice on how to fix her car. You explain that you don't have that type of expertise, but since she is in a library you say, “*Find a book* and figure it out on your own.”

The examples in (4)—which can be enriched with distinctions such as requests, exhortations, prohibitions, etc., and which can be understood as falling in one of the subtypes identified—illustrate that imperatives can be used to realize quite different speech acts. There is a lot of discussion about the types of speech acts expressed by imperatives in the semantics and pragmatics literature (see Portner, 2005; Kaufmann, 2012 for recent overviews), and often the question is raised whether the imperative has a uniform meaning. The obvious variation illustrated above suggests that the imperative is flexible in illocutionary force, but it is also generally recognized that the command is the “prototypical” use of the imperative (as the word itself suggests). Considering these four imperative types, the command is relatively more important from the perspective of the speaker since it is the only speech act of the four driven primarily by the speaker's goal or needs. The speaker has authority and uses the imperative as a command to get the addressee to do something the speaker wants or deems necessary. The other three imperative types (explanation, permission, and advice) take the perspective of the addressee, and involve primarily addressee goals, i.e., the imperative is used to further a goal of the addressee. By their very nature, then, addressee-goal imperatives appear weaker. This difference has not been featured prominently in the literature, but it is instrumental, as it turns out, to understanding the distinctive pattern of command we observe in our studies.

The different types of imperative speech acts are derived at the semantics/pragmatics interface on the basis of different contextual conditions^2^ Framing our observations in terms of preference (Condoravdi and Lauer, 2012), in commands such as (4a), the speaker has a very strong preference for the addressee to find a book, and the addressee knows that he/she is responsible for the realization of the preference. Something similar holds for explanations, although it is in the interest of the addressee (rather than the speaker) to follow through. In permissions such as (4c), it is not the speaker but the addressee who has a preference to find a book. In this context, the imperative expresses a change of the speaker preference to the preference of the addressee. Likewise, in speech acts of advice such as (4d), the speaker either has a weak preference for the addressee to find a book or the speaker may add the preference of the addressee to find a book to his/her preference. Either way, the speaker does not have a strong preference for the proposition expressed by an imperative of advice and addresses only the goal of the addressee.

Imperatives may thus be thought as having a uniform semantic core of preference, but can be used to convey various speech acts depending on contextual conditions. Chief among those acts is the act of command, which relies on the speaker's goal to make the addressee bring about action to achieve that goal. In this sense, the command reveals the strongest force of the imperative, since the speaker is personally invested in having their goal realized. The other three types we distinguished are rooted in the perspective of the addressee, hence the speaker is less invested in their realization—they can therefore be seen as weaker from the speaker's perspective. In other words, we can view the four types in (4) as realizing a two-way distinction based on speaker perspective and strength: speaker-oriented imperatives (command, strong), versus addressee-oriented imperatives (weaker from the speaker's perspective). The latter category is the one that involves more variability in illocutionary force, it is therefore not unreasonable to expect more variability in the means of realization.

Non-manual marking of imperative speech acts expresses important pragmatic information that can be used to specify the particular act expressed with an imperative. Although these non-manual markings are not totally conventionalized (cf. Donati et al., 2017), we assume that the different uses of imperatives can be understood by prosodic cues alone. In the three studies presented here, we ask how strongly and how consistently the pragmatic differences, i.e. the illocutionary forces of the imperatives in (4), are encoded in ASL and understood by ASL signers and three other groups of signers and non-signers without exposure to ASL.

With regard to comprehension, we are interested in determining across groups whether the cues for the imperative types show specific groupings—e.g., speaker- (command) vs. addressee-oriented imperatives (permission, advice, explanation), or, “must” type imperatives (command, explanation) vs. “may” type imperatives (permission, advice). With regard to the groups, we entertain two hypotheses, which may seem like they are competing, but we expect the results to support both of them, at least to some extent. The Hypothesis of Universality (Hypothesis A) predicts that the cues marking of pragmatic distinctions in imperatives reflects universal expressive strategies based on facial expressions, such as those described in Ekman and Friesen (1971). If this is the case, the meanings should be accessible to all of the groups in our studies equally, and knowledge of ASL should not provide any advantage. The Hypothesis of Arbitrariness (Hypothesis B) predicts that the cues marking of pragmatic distinctions in imperatives are entirely arbitrary and language-specific, and the meanings should not be accessible to anyone without knowledge of ASL grammar. We expect the results to support both hypotheses to some extent, since we not only expect the facial expressions marking imperatives to be accessible to all groups, but also that their particular grammatical distribution in ASL grammar will offer a significant advantage to ASL signers in distinguishing among imperative types. Study 1 involves the production of five conditions (the four types of imperatives mentioned above and neutral sentences) by a Deaf native ASL signer. We annotate and analyze several different prosodic cues for scope and quantity across the five conditions. In Studies 2-3, we then use these production data as stimuli in a task designed to study the comprehension of the speech acts corresponding to the five conditions. Study 2 examines their comprehension by other Deaf native and early learners of ASL. Study 3 expands the groups of participants performing the comprehension task to include a group of Deaf DGS signers, and two groups of non-signers: a group from the United States and a group from Germany. All three studies were approved and carried out in accordance with the recommendations of the Internal Review Board of the University of Chicago for the ethical treatment of human subjects, and with written informed consent from all subjects (IRB protocol 14-0410). All subjects gave written informed consent in accordance with the Declaration of Helsinki.

## Study 1

### Materials and methods

#### Participants

The sentences were produced by one Deaf, third-generation, native ASL signer (male, age 36).

#### Stimuli

All items consisted of two signs, which were combinations of four verbs consisting of a single path movement—pick/find (these signs are homophonous), take, throw-away, and keep—and four nouns consisting of a 2-movement reduplicated sign—book, hat, paper, and watch. Each sentence appeared in five conditions: neutral, command, explanation, permission, and advice (16 sentences × 5 conditions = 80 items). The number of words and syllables per sentence was therefore uniform across items, as was word order. Verb+Noun_DO_ is the unmarked order for all sentences employed. The neutral clause was extracted from the sentence frame “i like” to ensure a neutral production (i.e., a declarative sentence expressing assertion).

#### Procedures

Definitions and instructions were given in ASL. The signer was told that the task was about understanding the meaning of ASL sentences. After providing the signer with definitions of the imperative types and examples of contexts in which each of the speech acts would be produced, such as those in (4), he was instructed to construct an imagined context to achieve the targeted imperative type for each item presented. A set of 8–10 practice items were then presented. After the experimenter was satisfied that the signer understood the task and was comfortable with it, the 80 items were each presented in pseudo-random order using a Powerpoint presentation. The signer could control the pace of presentation. The types of imperatives were prompted by a static image of a sign for the five types of sentences—neutral, command, explanation, permission, and advice—followed by static images of the two signs making up the sentence. The signer was allowed to repeat the sentences until he was satisfied with the production for each item. The clips he judged to be the most representative for each item were then clipped and annotated in ELAN (https://tla.mpi.nl/tools/tla-tools/elan/). The annotations were completed by a research assistant who is a fluent second language learner of ASL and was trained in annotating non-manual cues. 20% were re-annotated by the first author with reliability of 95% for cues, and 90% for the duration of each sign. After discussion all discrepancies were resolved.

The cues that were analyzed are listed and defined in Table 1, and these were annotated for the verb and the noun separately. The manual cues were sign duration and hold duration, and the non-manual cues were head nod, head tilt, mouthing, and eyes wide. In keeping with the distinction we made in the introduction, annotators were sensitive to the possible use of “expressive” and “iconic” non-manuals. We wanted to analyze focus on expressives in this paper, so we constructed stimuli with simple verbs and nouns that were not prone to iconic non-manuals. As predicted, we found no manner, size, or shape non-manuals in the signer's productions.

**Table 1:** Prosodic cues analyzed in the productions of imperatives.

**Property**	**Definition**
1. Sign duration	Length of time from full formation of initial handshape to initial decay of final handshape; this measure includes sign-final holds
2. Hold duration	Periods during which the handshape and location of the sign were static
3. Head nod	Continuous nodding of the head during the production of a sign
4. Head tilt	Tilts of the head backward, forward, or sideways
5. Mouthing	The silent production of some or all of the corresponding English word
6. Eyes wide	Eyes more widely open, accompanied by a penetrating gaze

The set of cues included in the analysis was arrived by first annotating a much larger set of cues that are associated with intonational phrases and have been observed in the literature (Nespor and Sandler, 1999; Brentari and Crossley, 2002; Pfau and Quer, 2010; Brentari et al., 2011, 2015; Sandler, 2012). In addition to the six cues in Table 1, we annotated transition duration between signs, brow raise, brow furrow, body lean, squint, single head nods, smile, and corners of the mouth turned down, but the cues in this last set were used too rarely or showed no relevant pattern, and so were not included in the analysis. We then added cues that we saw in the data that were previously unattested. We added eyes wide to characterize a very open eye position accompanied by a penetrating eye gaze that appeared frequently in these data.

Examples are given in (5) of one sentence across all conditions with its annotated cues; the distribution of cues is presented in the Results section. Sign duration is noted by adjusting the space between the glosses. Since these cues are relative and dynamic, video examples are provided in the Supplementary Materials.

(5) Example ASL sentences (See also Supplementary Materials, Videos 5–9) 
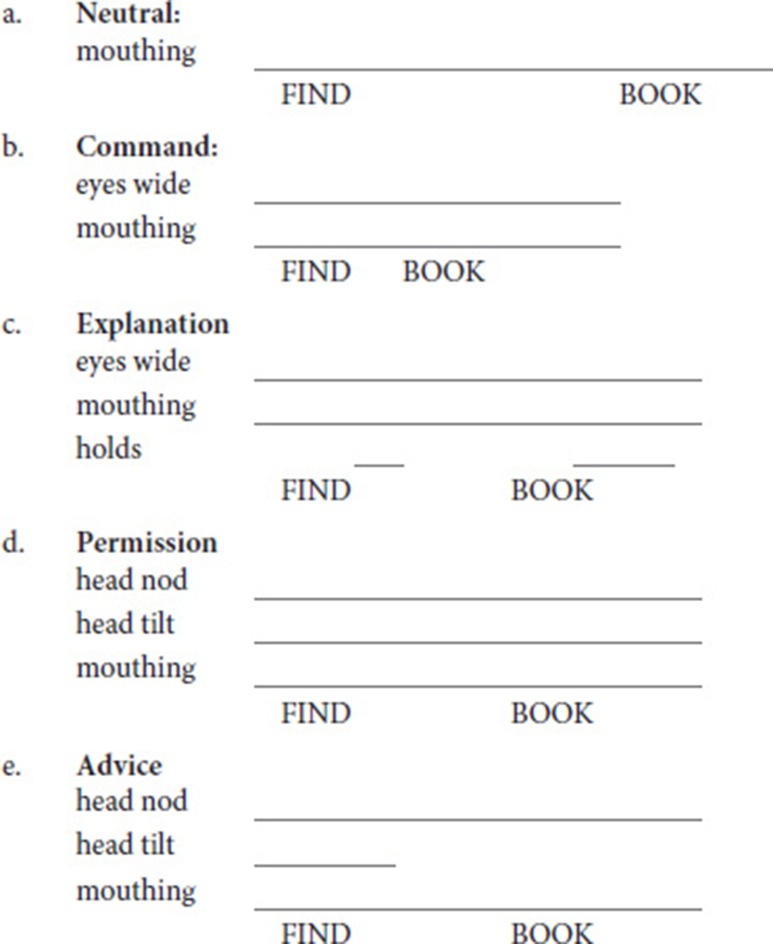

### Results

**Figure 1:**
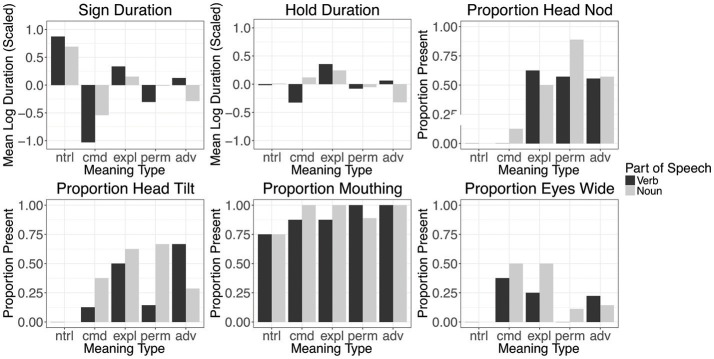
The distribution of the six prosodic cues annotated in this study. For the temporal cues (sign duration and hold duration) the scaled, log-transformed durations are plotted against the average value across all conditions (assigned the 0 value). For the non-manual cues proportions are provided.

We analyzed each of the cues with regard to its use on the verb and on the noun in the 80 sentences. The distribution of each cue across conditions is given in Figure 1. For the temporal cues (sign duration and hold duration), we applied a log-transformation to the values, and then scaled the log-transformed durations to have a mean of 0 and a standard deviation of 1. We then report the average scaled duration for each meaning type. Thus, values below 0 indicate shorter durations than the overall average across all conditions, and values above 0 indicate longer durations than the average across all conditions. For the remaining cues, we report the proportion of signs that expressed each cue at any point during the sign.

A summary of our findings is as follows. The average sign duration is longer in neutral sentences than all other conditions, and shorter in commands than in all other sentence types. Hold duration has more modest effects, but sentences of explanation have longer than average holds on both signs, while commands have shorter holds on the verb, and sentences of advice have shorter holds on the nouns. Sentences of explanation, permission, and advice all employ head nod to some degree, and sentences of permission have an increased use of head nods on the noun; in contrast, neutral sentences and commands rarely use this cue. Head tilts also occur with sentences of explanation, permission, and advice more frequently than with neutral sentences or commands; they are more likely to appear on the verb in sentences of advice, on the noun in sentences of permission, and on both in explanations. Mouthings accompany exclusively the verbs and nouns; no other mouthing or mouth gestures occurred. Mouthings occur quite frequently, but are less frequent in neutral sentences. The cue eyes wide appears most frequently in commands, and also appears frequently on the nouns of explanations.

We used a multinomial logistic regression model on the cues to try to predict the condition. We used 4-fold cross-validation to assess the accuracy of the model. The data are randomly split into 4 segments, and the model is trained on each possible set of three segments and then used to predict the remaining segment. We then compute the accuracy of these predictions. Because the model is predicting across five conditions, a baseline chance performance is 0.20.

Neutral sentences and commands are predicted well above chance, and as presented in Table 2 they are rarely mistaken for other sentence types. Explanation, permission, and advice sentences are rarely mistaken for other meaning types, but they are frequently mistaken for one other. From among these three types, permission is predicted most accurately and it is mistaken for other meaning types relatively least often, whereas sentences of advice and explanation are often mistaken for one another.

**Table 2:** Results of the Logistic regression model for Study 1 (Production task).

**Stimulus**	**Neutral**	**command**	**Explanation**	**Permission**	**Advice**
**MODEL OUTPUT**					
Neutral	**0.81**	0	0.06	0	0.12
Command	0	**0.62**	0.19	0.12	0.06
Explanation	0	0.12	**0.38**	0.25	0.25
Permission	0	0.12	0.25	**0.5**	0.12
Advice	0	0.06	0.44	0.19	**0.31**

*The numbers in bold indicate accurate responses*.

### Discussion

From the analyses above we can arrive at several generalizations concerning the distribution of cues. This can be schematized as in (6).

(6) Reliability of prediction of meaning types based on the regression model
  Neutral > Command > Permission > Explanation, Advice

Neutral sentences could be identified as distinct from any of the imperative types because they displayed the fewest non-manual cues, both in type and frequency. Neutral sentences also had longer average sign durations than any of the imperatives. In essence, the lack of non-manual cues and the relatively long durations were rather strong indications that the sentence is a neutral sentence. It has been shown that the presence and the *absence* of cues may be informative for sentence meaning (Herrmann, 2015).

Of the imperative sentence types, commands could be identified by the non-manual cue of eyes wide, along with shorter sign durations. Commands were also less likely to have head nods, head tilts, and the holds on verbs were shorter than average. Explanations had longer holds on both signs, and sentences of advice had shorter than average holds on the noun, but these effects were relatively modest. The imperative findings in Study 1 suggest clearly a pattern of commands versus the rest, and this divide maps onto the notion of speaker goal vs. addressee goal outlined in the theoretical work on these speech act types.

Addressee goal imperatives such as advice, explanation, and permission do not clearly have unique prosodic patterns. The cues that appear on these sentence types were subtle distinctions of distribution, and sometimes appeared on only one of the two signs—either the verb or the noun. For example, sentences of advice and permission both used head tilts but advice was more likely to have this cue on the verb, and permission more likely to have it on the noun. From among sentences of advice, explanation, and permission, sentences of permission are predicted more reliably than those of advice and explanation. One interpretation of the variability would be that all items without a clear absence or strong presence of prosodic markers are unclassified. Another interpretation is in agreement with the weaker nature of those imperatives, i.e., weaker in the sense that the speaker is less invested in their realization (as noted earlier). Given that the addressee's investment is variable, the observed flexibility is expected.

The regression model provides some predictions about what humans might attend to in evaluating these sentences. With regard to the type of cues, we expected both temporal and non-manual cues to differentiate these meaning types and, indeed, that is what we found. Since our sentences were from one signer, we cannot rule out that other cues may also fill these same roles, or that a pattern of general prominence is also factor, in addition to the specific cues we found here. We now turn to the two studies of comprehension of these cues by a group of signers of ASL (Study 2) and by three other groups (Study 3): DGS signers from Germany, non-signers from the USA, and non-signers from Germany. These studies will help us understand which cues employed to identify these meaning types are accessible to the different groups.

## Study 2

Study 1 has informed us about the strength and frequency of a set of six prosodic cues and their patterning in the five target meanings, but they do not address whether the regression model is predictive of the comprehension of these meanings by ASL signers, nor what other cues may influence ASL signer judgments. For example, the degree of tension in the body, and movement acceleration and deceleration, are both noticeable, but we could not reliably annotate these cues from the video stimuli; therefore, the results of this study will help us determine if the cues we annotated are indeed those that signers attend to when identifying these five types of meanings based on prosody.

### Materials and methods

#### Participants

Thirteen adult, Deaf native or early learners of ASL signers from the United States participated in this study. Eight participants learned ASL from birth, while five participants were early learners who acquired ASL prior to age 5 (eight females; five males). The ages of our participants were as follows: three were 18–25 years, two were 25–35 years, four were 35–45 years, one was 45–55 years, two were 55-65 years, and one was over 65 years.

#### Stimuli

The stimuli consisted of the 80 sentences analyzed in Study 1. A sample video for each of the meaning types is provided in the Supplemental Materials.

#### Procedures

Using a web-based interface, participants completed both a multiple choice and a matching task. In this paper, we analyze only the multiple-choice task^3^. The instructions for the task and definitions of the meaning types and sample contexts were presented in ASL on videotape to ensure consistency across participants, along with some English text to label the conditions and sentence choice options. The participants watched the instructions before proceeding to two practice sentences using commands and neutral sentences. The 80 two-sign sentences were presented in pseudo-random order, and signers were free to return to the definitions and sample contexts as often as needed. The 80 sentences were split evenly between the multiple choice and the matching tasks, with half of the participants completing the multiple choice task first and half of the participants completing the matching task first. For the multiple choice task, each item consisted of a slide containing the video and 5 multiple choice buttons with labels corresponding to the meaning type. Participants were asked to pick the meaning that they thought was being expressed. The items were presented in blocks of 10.

### Results

The accuracy and confusion matrices are provided in Table 3. The results are strikingly similar to the predictions of the regression model of Study 1, with comparable confusions among the same imperative types. ASL signers were even better at identifying neutral sentences and commands than the regression model predicted in Study 1 (0.93 and 0.82). Explanation, permission, and advice are about as accurate as would be predicted by the model and confusable in the same ways. Among the group that includes explanation, permission, and advice, sentences of permission are slightly easier to predict (0.50).

**Table 3:** Accuracy and confusion matrices for Study 2 (Comprehension-ASL signer group).

**Condition**	**Neutral**	**Command**	**Explanation**	**Permission**	**Advice**
**ASL SIGNERS' RESPONSES**					
Neutral	**0.93**	0.00	0.03	0.02	0.02
Command	0.01	**0.82**	0.07	0.02	0.09
Explanation	0.06	0.01	**0.35**	0.24	0.35
Permission	0.02	0.00	0.15	**0.50**	0.33
Advice	0.05	0.04	0.26	0.32	**0.34**

*The numbers in bold indicate accurate responses*.

### Discussion

Because the results from the Deaf native ASL signers are so similar to the regression model results, we can be reasonably certain that we have annotated most of the relevant cues that ASL signers employ to make their judgments of these five meanings. We can also say with some certainty that ASL signers are—as expected—able to identify speech acts on the basis of temporal and non-manual prosodic cues.

## Study 3

We now turn to the question of the accessibility of the ASL prosodic cues used for imperatives by three additional groups using the same comprehension task as was used in Study 2. The groups are: signers of DGS, non-signers from the United States, and non-signers from Germany. The DGS signers' results will inform us about how accessible the meanings of the prosodic cue patterns are to people without exposure to ASL, but with knowledge of a sign language and who are accustomed to attending to the hands and face for prosodic cues. The results of the American, hearing non-signers inform us about accessibility that might be due to shared gestural competence based on residing in the same country. The results of the German, hearing non-signers will inform us about broader accessibility of these patterns of prosodic cues, at least extending to communities whose origin is Western Europe.

Questions of accessibility also indirectly address the issue of how these cues come to be conventionalized, especially because the imperatives utilize facial expressions of emotions and mental states.

### Materials and methods

#### Participants

The participants in this study consisted of three groups. Group 1 consisted of fifteen adult, Deaf native or early learners of DGS from the Göttingen area who had no knowledge of ASL. Ten participants learned DGS from birth, and five participants were early learners who acquired DGS prior to age 7 (9 female; 6 males). The ages of our participants were as follows: four were 18–25 years, three were 25–35 years, one was 35–45 years, four were 45–55 years, two were 55–65 years, and one was over 65 years.

Group 2 consisted of 17 hearing American non-signers (recruited through Amazon Mechanical Turk) who had no knowledge of any sign language (7 females; 10 males). The ages of our participants were as follows: three were 18–25 years, nine were 25–35 years, and five were 35–45 years.

Group 3 consisted of 15 German non-signers who had no knowledge of any sign language (5 females; 10 males). The ages of our participants were as follows: four were 18-25 years, and 11 were 25-35 years.

#### Stimuli

The stimuli consisted of the 80 sentences analyzed in Study 1. Like the American signers, the American non-signers completed both a matching and a multiple-choice task. The German signers and non-signers performed the multiple-choice task for all 80 sentences. A sample video for representative sentences for each of the meaning types is provided in (5) and in the Supplementary Materials.

#### Procedures

Instructions, definitions and contexts for the American non-signers were translated from ASL into English and presented as English text. The instructions, definitions and contexts for the German non-signers were translated from English into German and presented in German text. The instructions, definitions and contexts for the DGS group were translated from German into DGS, videotaped and presented in DGS with some German text to label the buttons, etc., parallel to the ASL instructions of Study 2. The other procedures for Study 3 were the same as for Study 2.

### Results

The accuracy and confusion matrices for all three groups are provided in Table 4. There are two main results. First, the ASL signers from Study 2 as a group performed better than the other three groups, and second, the three non-ASL groups performed similarly to both the predictions of the regression model of Study 1, and to the ASL signer comprehension results in Study 2. Like the ASL signers, these three groups were better at identifying neutral sentences and commands, and they had less accuracy and more confusion in identifying sentences of explanation, permission, and advice.

**Table 4:** Accuracy and confusion matrices for the DGS, American non-signer, and German non-signer groups.

**Stimulus**	**Neutral**	**Command**	**Explanation**	**Permission**	**Advice**
**DGS SIGNERS' RESPONSES**					
Neutral	**0.78**	0.05	0.06	0.06	0.04
Command	0.02	**0.75**	0.11	0.07	0.05
Explanation	0.08	0.03	**0.32**	0.2	0.37
Permission	0.05	0.06	0.19	**0.37**	0.33
Advice	0.05	0.12	0.22	0.29	**0.32**
**AMERICAN NON-SIGNERS' RESPONSES**					
Neutral	**0.78**	0.02	0.13	0.03	0.04
Command	0.01	**0.76**	0.16	0.01	0.05
Explanation	0.06	0.11	**0.29**	0.24	0.30
Permission	0.04	0.05	0.26	**0.44**	0.21
Advice	0.05	0.13	0.29	0.26	**0.26**
**GERMAN NON-SIGNERS' RESPONSES**					
Neutral	**0.83**	0.02	0.08	0.04	0.03
Command	0.03	**0.67**	0.16	0.04	0.11
Explanation	0.08	0.06	**0.41**	0.18	0.27
Permission	0.05	0.06	0.26	**0.35**	0.29
Advice	0.06	0.08	0.33	0.27	**0.26**

*The numbers in bold indicate accurate responses*.

In order to confirm these impressions, we also used a logistic regression model that predicts whether participants gave the correct response on each item. We included meaning type and participant group as predictors, as well as the interaction between these terms (in case some group performs significantly better or worse on a particular meaning type). We used stepwise regression with the Bayesian information criterion (BIC) to select relevant predictors, since there are a large number of interactions for the size of our data set.

The results of the logistic regression model after variable selection are presented in Table 5. Note that the ASL-signing participants and neutral sentences are the baseline encoded in the intercept for the model. Positive coefficients mean better performance than the baseline, whereas negative coefficients mean worse performance than the baseline.

**Table 5:** Results of the Logistic regression model for Study 3 (Comprehension task-all groups).

	**Estimate**	**Std. Error**	***t*-value**	**Pr(>|t|)**
(Intercept)	0.9	0.025	36.06	<2e-16^***^
Dgs	−0.08	0.024	3.29	0.00099^***^
German	−0.11	0.025	4.47	8.0e-06^***^
American	−0.08	0.026	3.02	0.0025^**^
Command	−0.09	0.024	3.59	0.00034^***^
Explanation	−0.52	0.027	−19.45	<2e-16^***^
Permission	−0.43	0.024	−18.24	<2e-16^***^
Advice	−0.53	0.023	−22.06	<2e-16^***^
(Interaction) German × explanation	0.15	0.04	3.66	0.00026^***^

** means ≤ 0.01;

*** *means ≤ 0.001*.

The negative coefficients for all three groups show that they achieve lower accuracy than the ASL-signing participants. There is no statistically discernible difference between the coefficients for each group. Additionally, the only interaction that was selected was the interaction of hearing German group and explanations, with hearing German participants identifying explanations significantly better than the other groups. Aside from this one difference, this shows that the three groups have a pattern of performance that is largely the same in terms of overall accuracy, as well as their accuracy with regard to the meaning types: neutral sentences were most accurately identified, then commands, and then the other three meaning types, with permission the best identified of these three types for DGS signers and American non-signers, and advice the worst identified type for German non-signers.

All of the sentence types and all of the groups were selected as significant predictors. Commands have a small but significant negative coefficient, showing that participants are slightly less accurate at identifying this type than neutral sentences. Explanation, permission, and advice all have much greater negative coefficients. There is no statistically discernible difference between the coefficients for explanation and advice, suggesting comparable performance on these sentence types. However, the permission coefficient is slightly smaller, suggesting better accuracy for this meaning type.

**Figure 2:**
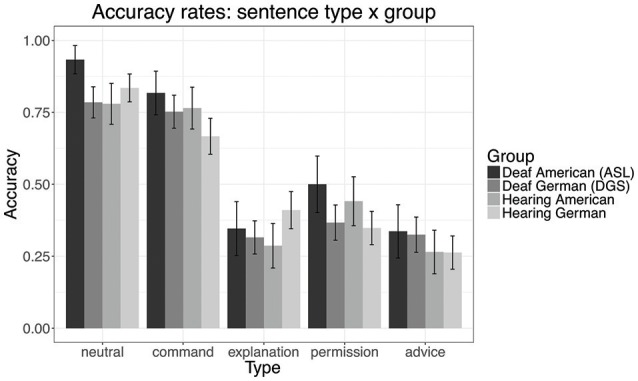
Accuracy rate reported for meaning type by group, with standard error bars.

These patterns can also be seen in the graph below in Figure 2, which shows the accuracy rates by sentence type and group. The interval bars represent 95% confidence intervals for the proportion of correct responses.

## Discussion: study 3 and general discussion

Non-manual and temporal prosodic cues of a sign language can be used to distinguish certain speech acts, but the patterns for others are highly confusable. Our results across groups show that commands are distinct among imperative types in that they are the most easily identified type. This result is in agreement with our earlier establishing of command as the strongest (thus most proto-typical) speech act, because the speaker is highly invested in the action she wants the addressee to do. We predicted in our earlier discussion, and indeed found here, a pattern distinguishing two classes of imperatives—commands versus the rest—mapping onto speaker goal vs. addressee goal imperatives (Condoravdi and Lauer, 2012). The results of our study are thus in line with recent analyses of the semantics and pragmatics of imperatives, and the core distinction we drew in section Imperatives.

Addressee goal imperatives such as advice, explanation, and permission were found to not have unique prosodic patterns. The cues that appear on these sentence types were subtle distinctions of distribution, and sometimes appeared on only one of the two signs—either the verb or the noun. For example, advice and permission both used head tilts but sentences of advice were more likely to have this cue on the verb, and sentences of permission were more likely to have it on the noun. The variability is in agreement with the weaker nature of those imperatives, i.e. weaker in the sense that the speaker is less invested in their realization.

Imperative meanings are accessible to people without exposure to ASL; however, as the cues and their distribution have been further conventionalized in ASL, the ASL signers perform better overall. Hence, the results of Studies 2 and 3 provide evidence that certain non-manual and temporal prosodic cues are integrated in the grammatical system of ASL as speech act indicating devices (for the grammaticalization of gestures, see van Loon et al., 2014).

Turning to our two hypotheses from the beginning of the paper we can conclude that both the Hypothesis of Universality and the Hypothesis of Arbitrariness are to some extent supported. The Hypothesis of Universality predicted that non-manual marking of pragmatic distinctions in imperatives reflects universal strategies for expressing mental states. We found that despite the fact that all groups found this task difficult, all were able to perform the task at above chance levels. The Hypothesis of Arbitrariness predicts that non-manual marking of pragmatic distinctions, such as different uses of imperatives, is entirely arbitrary and language-specific, and the meanings should not be accessible to anyone without knowledge of ASL. And, indeed, despite the fact that the content of the prosodic cues was accessible to non-ASL-signers, the additional knowledge of the patterns of conventionalization gave ASL signs a boost in performance that was significant.

Let us first address the similarities in performance across groups. There are at least two possible reasons why neutral sentence and commands are identified most easily, and sentences of explanation, permission, and advice are highly confusable. The first is the system of cue marking. Neutral sentences have the fewest cues and are, as expected, unmarked. Commands are accompanied by the highest number of cues, so their patterns are more structurally distinct from among these five meaning types. Sentences of explanation, permission, and advice have a more complex system of marking, and the differences among the cue patterns for these meanings are more subtle and less consistent; specifically, the same cues are used across all of them to some degree, appear on fewer of the sentences overall, and the differences among these sentences are rather small.

A second possible reason for the similarity in performance across groups is that the content of the prosodic cues is familiar to all groups, at least to some extent. Imperatives are associated with speech acts, which are associated with specific emotional and mental state facial expressions that accompany them in canonical contexts. Across groups cues involving mental states might include non-manual cues, such as a stern expression for commands, differing degrees of an inviting expression for advice and permission, as well as specific timing cues, such as a slower articulation for explanation. Moreover, commands demand something of the interlocutor and have more negative valence, while the other three imperative types are offering something to the interlocutor and have more positive valence. These results are in line with the assumptions that properties of the context are relevant to specify the speech act performed with an imperative. The interaction of an underspecified imperative sentence mood with specific pragmatic conditions yields the speech act expressed in a specific contextual setting. In this context, the non-manual and temporal prosodic cues seem to function as speech act indicating devices.

Ongoing pilot data by our team involves two follow-up studies using a set of English sentences that parallels the ASL sentences (Brentari et al., 2017) and suggests that some of the same facial expressions are used in English co-speech gesture and in ASL. In preliminary analyses we found that head tilts were used in English sentences of permission and advice, similar to their use in ASL. Some of the temporal cues had parallel realizations as well; for example, from among the imperatives, explanations tended to be longer than any other imperative type in English, and in ASL as we have seen here, perhaps because of their pedagogical nature.

Kuhn and Chemla (2017) and Domaneschi et al. (2017) provide further evidence that hearing non-signers use facial expressions to indicate various speech acts. Kuhn and Chemla (2017) presented non-signers with four emblematic gestures used in American culture combined with facial expressions indicating four conditions. Expression of assertion, wh-question, yes/no question, and command were combined with thumbs up (“good”), thumb pointing (“him”), wrist tap (“time”), and finger rub (“money”) gestures. For example, for the “money” theme, the possible sentences expressing the speech acts were: *It's expensive*. (assertion), *Pay up!* (command), *How much is it?* (wh-question), *Do you need money?* (yes/no question). Non-signers were able to match the condition with the facial expression at above chance levels. Likewise, Domaneschi et al. (2017) show that Italian speakers associate certain facial expressions with interrogative and directive speech acts. In particular, action units 1 and 4 indicate questions and action units 4 and 5 commands. As opposed to questions and commands, assertions are not marked by facial expressions. Hence, these studies provide evidence that paralinguistic facial expressions may contribute to the understanding of speech acts in spoken languages.

We now turn to possible explanations for the difference between comprehension accuracy in ASL signers vs. the other three groups. Despite the fact that the DGS signers and non-signers can perform this task at above chance levels, the ASL signers were significantly better. Given the result that ASL signers are significantly more accurate on the comprehension task than the other three groups, the ASL signers are more sensitive to the combinatorial properties of these prosodic cues and to their temporal distribution than the other groups. This emphasizes that the grammar of a language concerns the distribution of forms as much as the content. Dachkovsky et al. (2013) have discussed a number of language-particular differences in the non-manual grammatical markers of ISL and ASL in ways that are relevant here. They outline the very subtle reasons for why ISL and ASL might demonstrate differences in the distribution of language-specific cues, both for phonetic and semantic reasons. For example, the two sign languages produced squints differently phonetically—ISL signers tighten their lower eyelids to produce a narrowed eye aperture, while ASL signers raise the cheeks to accomplish the same result. Semantically, the given-new distinction in the two languages both use squints, but with different frequency, based on how salient or accessible the information is to the interlocutors (Ariel, 2001). ASL signers use squint to mark given information only when that information is very low in “givenness” (low accessibility), while ISL uses it at both low and mid degrees of accessibility.

We acknowledge that our studies have a few weaknesses. One is that there was only one ASL signer for Study 1 and the production results and subsequent items in Studies 2 and 3 were based on his productions. It would be helpful to see whether the results of Studies 2 and 3 are due to the idiolectal cues of one signer or generalizable across signers. Another is that we did not offer alternatives to participants other than neutral and the four imperative types; we might have included a yes/no-question choice, for example. A third weakness is that, even though the 16 sentences appeared in all 5 meaning types and the lexical signs were not sufficient to arrive at the meaning type, the ASL signers knew the signs and they might have been processing the sentences somewhat differently than the other three groups. A follow-up study could rectify all three of these weaknesses by having more signers and additional groups engaged with different tasks—a yes/no, a multiple choice task, and perhaps even a matching task, and instead of ASL signs, also use nonce signs. This work is just a first step along this path.

## Conclusions

The studies presented in this paper have focused on imperative speech acts that were expressed via prosody alone. These prosodic cues signaling emotions and mental states are only partially grammaticalized. Their content is accessible to non-signers to a large extent, while further conventionalization of these cues via their distribution give ASL signers a positive advantage in identifying the imperative speech acts that we investigated. We would argue that using a consistent distribution in alignment, form, and function is an important step in creating a grammatical form. The content of the form may be accessible to non-signers, but as they become conventionalized, signers become more sensitive to them in a particular systematic distribution.

## Author contributions

DB designed the project, initiated the studies, and oversaw all aspects of data collection, analysis, and interpretation. She also wrote the first draft of the manuscript and consolidated the co-authors' contributions into the final manuscript. JF helped design the study, particularly the online task, did all of the statistical modeling, and assisted in the preparation of the manuscript. EV executed translation of tasks and instructions into German, supervised their translation into DGS, collected the data from the German groups, and assisted in the preparation of the manuscript. AH and MS utilized their physical facilities to carry out data collection in Göttingen, Germany. AG, AH, and MS provided their respective expertise in semantics, non-manuals, and sign language grammars, and assisted in the preparation of the manuscript.

### Conflict of interest statement

The authors declare that the research was conducted in the absence of any commercial or financial relationships that could be construed as a potential conflict of interest. The reviewer, DS, and handling Editor declared their shared affiliation.

## Footnotes

^1^In addition, we wanted to study the temporal and non-manual markers of imperatives, and thus the sentences (i.e., the signs themselves and their order) had to be the same across all speech acts. As this is not the case for all types of imperatives, we avoided imperatives that involve different signs or different sign orders.

^2^We refer to the semantics/pragmatics interface together because we are concerned with sentence meaning that originates from sentence-internal factors (semantics) or from the surrounding discourse (pragmatics).

^3^We thought the matching task might be easier, but there was no statistical difference between types of items, so in Study 3, two of the three groups were given only multiple-choice items. To have comparable data for analysis across all groups we therefore analyzed only the multiple-choice items here.

## Supplementary material

Video 1 ASL assertion.

Video 2 ASL negation.

Video 3 ASL coordinate structure.

Video 4 ASL conditional structure.

Video 5 ASL neutral.

Video 6 ASL command.

Video 7 ASL explanation.

Video 8 ASL permission.

Video 9 ASL advice.
